# Unhealthy oral status contributes to the older patients with cognitive frailty: an analysis based on a 5-year database

**DOI:** 10.1186/s12877-022-03673-5

**Published:** 2022-12-19

**Authors:** Zhiqiong Jiang, Xintong Liu, Yang Lü

**Affiliations:** 1 Department of Geriatrics, The First Affiliated Hospital of Chongqing Medical University, 400016 Chongqing, China; 2 Department of Geriatrics, Affiliated Hospital of North Sichuan Medical College, 637000 Nanchong, China

**Keywords:** Cognitive frailty, Oral health, Physical frailty, Risk factor

## Abstract

**Background:**

Oral health is associated with the onset and deterioration of cognitive function and physical frailty, which can be improved with appropriate interventions. However, far too little attention has been paid to oral health status of elderly with cognitive frailty. The objective of this study was to investigate the oral health status and potential risk factors of elderly hospitalized patients aged 60 years or older with cognitive frailty.

**Methods:**

The participants’ assessment data derived from the Comprehensive Geriatric Assessment Database of hospitalized patients from The First Affiliated Hospital of Chongqing Medical University. Data were collected from April 2016 to December 2021. All participants underwent a face-to-face assessment conducted by professional evaluators. Physical frailty was defined by Fried’s criteria. Cognitive function was assessed by Mini Mental State Examination (MMSE). The cognitive frailty is characterized by the simultaneous presence of at least 1 Fried’s criteria and mild cognitive impairment according to Diagnostic and Statistical Manual of Mental Disorders 5th edition. The oral health was assessed according to 10-item Brief Oral Health Status Examination (BOHSE). The general demographic characteristics, BOHSE scores were compared between the cognitive frailty and non-cognitive frailty (control group). The score of BOHSE and ten items were included in the binary logistic regression analysis. The covariate characteristics were adjusted for a final model with a multivariate analysis.

**Results:**

A total of 425 patients (245 females) with cognitive frailty and 491 patients (283 females) with non-cognitive frailty were enrolled in this retrospective study. Univariate analysis showed statistically significant differences in age, education level, living arrangement, diabetes, Body Mass Index (BMI), Pittsburgh Sleep Quality Index (PSQI), depression between the two groups. The total BOHSE score of cognitive frailty was higher than that of the control group (4.35 ± 2.68 vs. 3.64 ± 2.60, Z = 4.07, *P* < 0.001). The average scores and the proportions of health changes and unhealthy states of tongue, mucosa tissue, gums, natural teeth, dentures, masticatory teeth and oral hygiene in cognitive frailty were greater than those of the control group (all *P* < 0.05). The binary logistical regression analysis showed that four or more natural teeth decayed or broken was independently associated with cognitive frailty after adjusting the age, gender, education level, living arrangement and BMI, PSQI, diabetes and depression (OR = 1.91, 95%CI: 1.20–3.07, *P* = 0.007). Additionally, while in the chewing position, those cases with a normal-occlusal-relationship number of less than 11 pairs had a higher risk of cognitive frailty than those with 12 pairs or more.

**Conclusions:**

The oral health status of older hospitalized patients over 60 years with cognitive frailty was worse than that of patients with non-cognitive frailty. But only four or more natural teeth decayed or broken and a reduction in chewing pairs were independent risk factors for cognitive frailty.

## Background

Age-related chronic diseases have become a global health concern and financial burden following the aging population growing. As a new target of active aging intervention, cognitive frailty has attracted extensive attention recently [1]. Cognitive frailty is a heterogeneous clinical manifestation characterized by the simultaneous presence of both physical frailty and cognitive impairment. The concept or operational definition of “cognitive frailty” was first proposed in 2013 by the international consensus group comprised of experts from the International Academy of Nutrition and Aging (IANA) and the International Association of Gerontology and Geriatrics (IAGG) [2]. Older adults with cognitive frailty have an increased risk of adverse health outcomes such as falls, disability, hospitalization, institutionalization, and 30-day mortality [3–7]. At the same time, a number of studies have confirmed that the risk of dementia transformation significantly increases in people with cognitive frailty, which is 2.3 to 5.58 times higher than that in people without cognitive frailty [8, 9]. Dementia cannot be reversed, but cognitive frailty can be reversed in some patients through appropriate intervention. Therefore, cognitive frailty can be used as an early intervention sign of dementia, making it a useful target for preventive measures against dependency in older people [10, 11].

Oral health, a basic part of individual physical and mental health, is one of the healthy standards of older persons, which has an important impact on the quality of life. The oral health by the WHO is a state of being free from chronic mouth and facial pain, oral and throat cancer, oral infection and ulcer, periodontal/gum disease, tooth decay and tooth loss, as well as abnormal conditions with restricted chewing, smile, psychosocial health and ability to speak. With the increase of age, the structure and function of the oral cavity of older adult could undergo aging changes, resulting in a series of oral health problems [12]. A number of studies have confirmed that oral health deterioration can affect cognitive and physical function in older people. For example, some studies suggested that tooth loss was independently associated with cognitive impairment or dementia, and risk of diminished cognitive function increased as incremental numbers of teeth lost. But timely prosthodontic treatment with dentures may reduce the progression of cognitive decline related to tooth loss [13, 14]. Meanwhile, poor oral status significantly predicted future physical frailty and disability. One recent study has found that the incidence of frailty increased by 5.0% for each tooth lost. Interestingly, older adults who use dentures are less likely to have musculoskeletal weakness, and have a better quality of life [15]. In a recent study, Zhang et al. reported that older adults who have more teeth are associated with a lower risk of cognitive frailty, but which does not take into account the mouth as a whole [16]. Their purpose is only to explore the association between number of teeth and cognitive frailty. Other oral health problems, such as chewing, oral hygiene, periodontal/gum disease, have not been reported in patients with cognitive frailty. In addition, it is not known whether oral health interventions can reverse cognitive function and physical weakness in patients with cognitive frailty.

As mentioned above, cognitive frailty, as an early intervention target for older health management, can be reversed by active cognitive training, exercises and so on (6,8,10,11). On the other hand, oral health is associated with the onset and deterioration of cognitive function and physical weakness, which can be improved with appropriate interventions [13–15]. However, far too little attention has been paid to oral health status of older hospitalized patients with cognitive frailty. In addition, whether oral health status assessment is related to cognitive frailty has remained unknown. Thus, the purpose of our study is to investigate the oral health status of older hospitalized patients with cognitive frailty, and examine the potential risk factors of oral health indicators for cognitive frailty.

## Methods

### Study participants

We have used the data from the Comprehensive Geriatric Assessment Database of hospitalized patients from The First Affiliated Hospital of Chongqing Medical University. Data were collected from April 2016 to December 2021. The inclusion criteria were as follows: (i) aged ≥ 60 years; (ii) Patients who are hospitalized with the medical order of the professional geriatrician and undergone the comprehensive geriatric assessment; (iii) The assessment data is complete; (iv) If the patient has undergone multiple assessments, data from the first assessment are taken. Exclusion criteria were as follows: (i) aged < 60 years; (ii) Patients with simple cognitive impairment or physical frailty; (iii) Patients with dementia; (iv) Patients with acute oral pain.

All participants underwent a face-to-face assessment conducted by professional evaluators. The evaluator has a postgraduate degree in nursing. She has obtained the qualification of comprehensive evaluator for the aged through training and has worked for more than twenty years. The evaluators learned oral health assessment under the guidance of an oral specialist. Data collation and statistical analysis were completed by two experienced doctors.

This retrospective study did not disclose personally identifiable data of any participants in any form. Hence, consent for participate is not applicable here. It was approved by the institutional ethics board of The First Affiliated Hospital of Chongqing Medical University (approved on 18 July 2012, No.15).

### Cognitive frailty definition and assessment

The diagnosis of cognitive frailty is based on the following diagnostic criteria proposed by the IANA and the IAGG in 2013: (i) presence of both physical frailty and cognitive impairment (CDR = 0.5), and (ii) exclusion of Alzheimer’s disease or other types of dementia. In our study, cognitive frailty is characterized by the simultaneous presence of at least 1 Fried’s criteria and mild cognitive impairment according to Diagnostic and Statistical Manual of Mental Disorders 5th edition.

#### Physical frailty assessment

Physical frailty is defined by Fried’s criteria. It can be assessed by using five components as follows: (i) unintentional weight loss, which is defined as involuntary weight loss over 5 kg in the previous one year; (ii) exhaustion, which is indicated by two self-reported questions drawn from the Center for Epidemiological Studies Depression scale. “How many times in the past week have you been struggling to get anything done?” and “How often do you feel unable to move forward?” With one or two answers of “often” or “most of the time” as exhaustion; (iii) weak muscle strength as evaluated by hand strength, which is measured by using electronic hand dynamometer (Zhongshan Camry Electronic Co. Ltd, Guangdong, China). Each participant took the standing position, held the handle of the dynamometer with the maximum force with the dominant hand, repeated the measurement twice, and recorded the maximum value. Grip strength of less than 28 kg for men or less than 18 kg for women was defined as reduced grip strength; (iv) Slowness. It is assessed using 6-meter fast gait speed test. A speed < 0.8 m/s indicates frailty-related slowness; and (v) Low physical activity. It corresponds to response “How do you usually do physical exercise?”. Responses like “no physical exercise” or “mostly sedentary” are indicative of low physical activity.

Then, each of mentioned-above five aspects was added as one point. Thus, participants were divided into three categories: frailty (three or more points), pre-frailty (one or two points) or robust (zero point). To improve the recognition of cognitive frailty, we also included patients in pre-frailty states.

#### Cognitive function assessment

Cognitive function was assessed by Mini Mental State Examination (MMSE). MMSE provides a comprehensive, accurate and rapid reflection of the subject’s intellectual status and degree of cognitive impairment. It includes the following seven dimensions: orientation to time, orientation to place, immediate memory, delayed memory, attention and computation, language and spatial vision. The 30-item MMSE score system ranges from 0 to 30, and a higher score indicates a better cognition. With respect to the cognitive intact participants, they were defined as those cases with MMSE of 27 or greater and without any clinical diagnosis of cognitive impairment. As for patients with MCI, the diagnostic criteria were according to Diagnostic and Statistical Manual of Mental Disorders 5th edition (DSM-5) published by American Psychiatric Association (2013): (i) MCI with subjective and objective examinations; (ii) cognitive decline in one or more aforementioned dimensions; (iii) unaffected daily living ability; (iv) absence of meeting diagnostic criteria for dementia; (v) exclusion of other diseases that may cause cognitive decline; and (vi) MMSE scores. The test scores of MMSE are related to the level of education, with cut-off points for the MMSE of 17 points for illiterate persons, 20 for primary school, 22 for junior school, and 23 for university or above education.

### Oral health status assessment

The oral health assessment is according to the Brief Oral Health Status Examination (BOHSE) (18–20). It consists of 10 items: lymph nodes, lips, tongue, mucous tissue, gums, saliva, natural teeth, dentures, masticatory teeth and oral hygiene. Each item is classified as graded 0 (healthy), 1 (changes) or 2 (unhealthy). About “natural teeth”, healthy means no decayed or broken teeth/roots, changes refer to 1–3 decayed or broken teeth/roots, unhealthy refers to 4 or more decayed or broken teeth/toots, or fewer than 4 teeth in either jaw. About “masticatory teeth” (including natural teeth and dentures), healthy means 12 or more pairs of teeth in chewing position, changes refer to 8 and 11 pairs of teeth in chewing position, unhealthy refers to 0 and 7 pairs of teeth in chewing position. The higher score refers to the worse oral health.

### Covariates

Sociodemographic characteristics were collected, including age, gender, education level, living arrangement, behavior (tobacco and alcohol), chronic disease (coronary heart diseases, hypertension, diabetes and osteoporosis) and chronic body pain. Depending on age, participants were divided into two age groups: young older (60–79 years), and old older (≥ 80 years). Education status was classified as illiterate, primary school, junior school and university or above. Living arrangement was classified three groups: with spouse or children, living alone and living in nursing homes. Participants were also divided into four groups based on body mass index (BMI) according to the World Health Organization Asian adult bodyweight standard: normal (18.5–24.9 kg/m^2^), underweight (< 18.5 kg/m^2^), overweight (25.0–29.9 kg/m^2^), and obese (≥ 30.0 kg/m^2^). The score of Geriatric Depression Scale-5 (GDS-5) ≥ 2 was considered likely to be depressed. Moreover, the score of Pittsburgh sleep quality index (PSQI) and chronic body pain were also collected.

### Statistical analysis

Differences were considered significant at *P* < 0.05. All statistical analyses were performed with SPSS version 20.0 (IBM Corporation, SPPS Inc, Chicago, IL).

#### Comparison of cognitive frailty and non-cognitive frailty

The continuous measurements are presented as mean ± SD or median (IQR) based on the data distribution. The categorical variables are shown as number and percentage (%). The t test or Wilcoxon non-parametric test was applied for normally or non-normally distributed continuous variables, respectively. Categorical variables were compared using Chi-square tests. The score of BOHSE was a continuous variable, but the score of each item could also be considered as a categorical variable. Thus, the non-parametric test and chi-square test were used to determine the differences of oral health status between cognitive frailty and control group.

#### Binary logistic regression analysis

The odds ratios (ORs) and 95% confidence intervals (CIs) were calculated using the multivariate logistic regression. Firstly, the cognitive frailty groups and control group were used as dependent variables, the score of BOHSE and ten items as independent variables were performed by binary Logistic regression in Model 1. In addition to gender, each variable with a *p*-value of < 0.05 in the t-test or chi-square test were adjusted as confounding factors for further analysis. Hence, age (0,<80; 1,≥80), sex (0, male; 1, female), education level (0, illiteracy; 1, primary school; 2, junior school; 3, university or above), living arrangement (0, with spouse or children; 1, live alone; 2, nursing home), diabetes (0, No; 1, Yes), BMI (0, 18.5–24.9; 1, < 18.5; 2, 25–29.9; 3, ≥ 30), PSQI (continuous), depression (0, No; 1, Yes) were considered as independent variables for the binary logistic regression analysis. Model 2 was adjusted for age, gender, education level, living arrangement and BMI; Model 3 was adjusted for age, gender, education level, living arrangement and BMI, PSQI, diabetes and depression.

## Results

### Participants

**Fig. 1:**
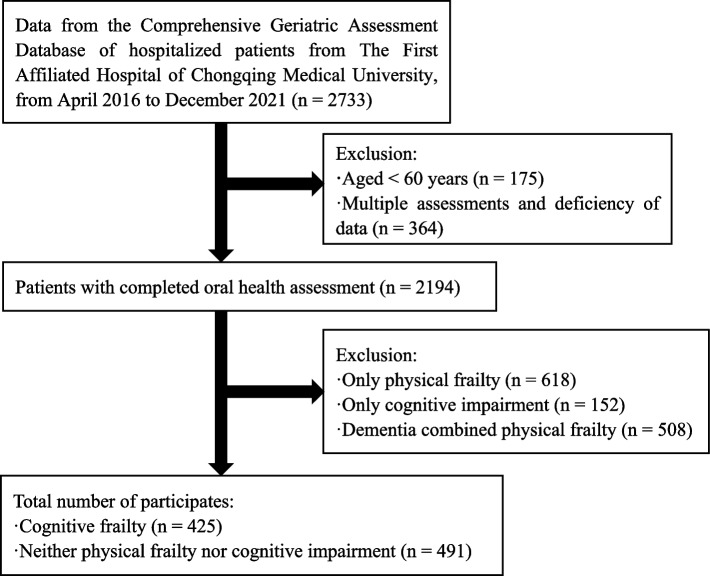
Flowchart of the participants included in the study

In the Comprehensive geriatric assessment database, a total of 2733 patients completed oral health assessment, excluding 175 patients younger than 60 years old, 364 patients with repeated measurements and incomplete data, and the remaining 2194 patients had complete assessment data. In these objects, 618 patients with only physical frailty, 152 patients with only cognitive impairment, and 508 patients with a clinician’s diagnosis of dementia and combined physical frailty were excluded. Eventually 425 patients (245 females) with cognitive frailty and 491 (283 females) controls without cognitive frailty (neither physical frailty nor cognitive impairment) were enrolled in this study (see Fig. 1).

### General characteristics of cognitive frailty and non-cognitive frailty

There were no statistically significant differences in gender, smoking and drinking, hypertension, coronary heart disease, osteoporosis and chronic body pain between two groups. However, the average age of patients with cognitive frailty was greater than that of control group, and the proportion of patients over 80 years old was higher in the cognitive frailty group. Compared with the control group, the proportion of illiteracy and primary school was higher in the cognitive frailty group, while the proportion of junior school and above was lower. The proportion of people with cognitive frailty living with their spouse or children was higher than those in the control group, and the proportion of people living alone and in nursing homes was higher. The proportion of diabetes in the cognitive frailty group was 36.5%, higher than 28.3% in the control group. The proportion of BMI in the normal range and overweight was lower in the cognitive frailty group than in the control group, while the proportion of both underweight and obese was higher in the cognitively frailty group (all *P* < 0.05) (see Table 1).

**Table 1:** General Characteristics of Cognitive Frailty and non-cognitive frailty

Parameters	Cognitive frailty (*N* = 425)	non-Cognitive frailty (*N* = 491)	Z/χ^2^	*P*
**Age, mean ± SD**	78.75 ± 7.46	71.78 ± 6.96	Z = 14.54	< 0.001
**Age, n (%)**			χ^2^ = 107.92	< 0.001
< 80	225 (52.9%)	415 (84.5%)		
≥ 80	200 (47.1%)	76 (15.5%)		
**Gender, n (%)**			χ^2^ = 0.00	0.998
Female	245 (57.6%)	283 (57.6%)		
Male	180 (42.4%)	208 (42.4%)		
**Education level, n (%)**			χ^2^ = 153.02	< 0.001
Illiteracy	49 (11.5%)	2 (0.4%)		
Primary school	142 (33.4%)	52 (10.6%)		
Junior school	176 (41.4%)	272 (55.4%)		
University or above	58 (13.6%)	165 (33.6%)		
**Living arrangement**			χ^2^ = 9.81	0.007
With spouse or children	351 (82.6%)	432 (88%)		
Living alone	51 (12%)	50 (10.2%)		
Nursing home	23 (5.4%)	9 (1.8%)		
**Smoking status, n (%)**			χ^2^ = 2.853	0.240
Never smoked	316 (74.4%)	388 (79%)		
Previous smoker	67 (15.8%)	65 (13.2%)		
Current smoker	42 (9.9%)	38 (7.7%)		
**Drinking status, n (%)**			χ^2^ = 2.46	0.292
Never drank	364 (85.6%)	417 (84.9%)		
Previous drinker	32 (7.5%)	29 (5.9%)		
Current drinking	29 (6.8%)	45 (9.2%)		
**Hypertension, n (%)**			χ^2^ = 0.00	0.993
Yes	252 (59.3%)	291 (59.3%)		
**Coronary heart diseases, n (%)**			χ^2^ = 0.24	0.624
Yes	152 (35.8%)	168 (34.2%)		
**Diabetes, n (%)**			χ^2^ = 6.96	0.008
Yes	155 (36.5%)	139 (28.3%)		
**Osteoporosis, n (%)**			χ^2^ = 2.47	0.116
Yes	117 (27.5%)	113 (23%)		
**BMI (kg/m** ^2^ **), n (%)**			χ^2^ = 15.20	0.002
< 18.5	42 (9.9%)	22 (4.5%)		
18.5–24.9	224 (52.7%)	289 (58.9%)		
25–29.9	130 (30.6%)	161 (32.8%)		
≥ 30	29 (6.8%)	19 (3.9%)		
**PSQI, median (IQR)**	11 (7, 16)	9.26 (5, 13)	Z = -4.914	< 0.001
**Depression**			χ^2^ = 57.22	< 0.001
Yes	135 (31.8%)	56 (11.4%)		
No	290 (68.2%)	435 (88.6%)		
**Chronic body pain**			χ^2^ = 2.46	0.117
Yes	241 (56.7%)	253 (51.5%)		
No	184 (43.3%)	238 (48.5%)		

*BMI* Body mass index, *PSQI* Pittsburgh Sleep Quality Index

### Oral health comparisons between cognitive Frailty and non-cognitive Frailty

#### Oral health score comparisons

**Fig. 2:**
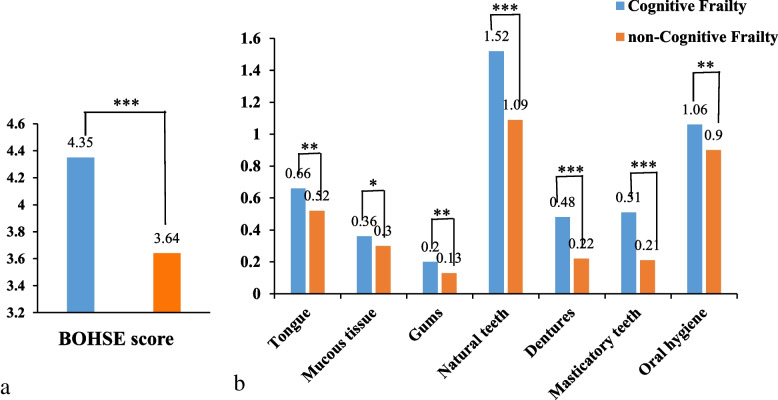
The comparison of the total BOHSE score (**a**) and scoring item comparisons in detail (**b**) between cognitive frailty and non-cognitive frailty

The total BOHSE score of cognitive frailty group was higher than that of control group (4.35 ± 2.68 vs. 3.64 ± 2.60, Z = 4.07, *P* < 0.001) (see Fig. 2a). The 10 scoring items of BOHSE were further compared in detail. Specifically, there were no statistically significant differences in the scores of lymph nodes, lips and saliva between two groups. As for the remaining seven items, the average score of each item was higher in cognitive frailty group (all *P* < 0.05) (see Fig. 2b).

#### Comparisons of categories of each item

The 10 indicators of oral health were further classified and compared according to their graded conditions. There were no statistically significant differences in the assessment of lymph nodes, lips and saliva between two groups. As for the remaining seven items, the proportions of health status of each item were higher in the control group than those in the cognitive frailty, whereas, the proportions of health changes and unhealthy states of each item were all greater in cognitive frailty than those in the control group (all *P* < 0.05) (see Table 2).

**Table 2:** Categories of BOHSE between Cognitive Frailty and non-Cognitive Frailty

Parameters	Cognitive frailty	non-Cognitive frailty	χ^2^	*P*
	(*N* = 425)	(*N* = 491)		
**Lymph nodes, n (%)**			χ^2^ = 1.74	0.188
Health	425 (100%)	489 (99.6%)		
Changes	0	2 (0.4%)		
Unhealthy	0	0		
**Lips, n (%)**			χ^2^ = 3.31	0.191
Health	262 (61.6%)	326 (66.4%)		
Changes	157 (36.9%)	162 (33.0%)		
Unhealthy	6 (1.4%)	3 (0.6%)		
**Tongue, n (%)**			χ^2^ = 11.53	0.003
Health	186 (43.8%)	267 (54.4%)		
Changes	198 (46.6%)	194 (39.5%)		
Unhealthy	41 (9.6%)	30 (6.1%)		
**Mucous tissue, n (%)**			χ^2^ = 6.12	0.047
Health	277 (65.2%)	348 (70.9%)		
Changes	141 (33.2%)	141 (28.7%)		
Unhealthy	7 (1.6%)	2 (0.4%)		
**Gums, n (%)**			χ^2^ = 8.00	0.018
Health	353 (83.1%)	439 (89.4%)		
Changes	59 (13.9%)	44 (9.0%)		
Unhealthy	13 (3.1%)	8 (1.6%)		
**Saliva, n (%)**			χ^2^ = 5.96	0.051
Health	229 (53.9%)	275 (56.0%)		
Changes	179 (42.1%)	209 (42.6%)		
Unhealthy	17 (4.0%)	7 (1.4%)		
**Natural teeth, n (%)**			χ^2^ = 68.04	< 0.001
Health	50 (11.8%)	145 (29.5%)		
Changes	103 (24.2%)	157 (32%)		
Unhealthy	272 (64%)	189 (38.5%)		
**Dentures, n (%)**			χ^2^ = 32.78	< 0.001
Health	312 (73.4%)	433 (88.2%)		
Changes	30 (7.1%)	16 (3.3%)		
Unhealthy	83 (19.5%)	42 (8.6%)		
**Masticatory teeth, n (%)**			χ^2^ = 52.40	< 0.001
Health	268 (63.1%)	406 (82.7%)		
Changes	98 (23.1%)	68 (13.8%)		
Unhealthy	59 (13.9%)	17 (3.5%)		
**Oral hygiene, n (%)**			χ^2^ = 10.42	0.005
Health	147 (34.6%)	221 (45%)		
Changes	106 (24.9%)	99 (20.2%)		
Unhealthy	172 (40.5%)	171 (34.8%)		

### Correlation analyses between oral health and cognitive Frailty

The score of BOHSE and ten items were included in the binary logistic regression analysis. The results showed that the health changes or unhealthy status in tongue, natural teeth and masticatory teeth were independently correlated with the cognitive frailty (Table 3, Model 1). Then, the age, gender, education level, living arrangement, BMI, diabetes, PSQI and depression were adjusted as confounding factors. After adjusted, the health changes or unhealthy status in tongue were no longer as a risk factor for cognitive frailty. Importantly, the indicator of four or more natural teeth decayed or broken was independently associated with cognitive frailty (OR = 1.91, 95%CI: 1.20–3.07, *P* = 0.007). Additionally, while in the chewing position, those cases with a normal-occlusal-relationship number of less than 7 pairs (OR = 2.62, 95%CI: 1.34–5.11, *P* = 0.005), or between 8 and 11 pairs (OR = 1.72, 95%CI: 1.14–2.66, *P* = 0.014), had a higher risk of cognitive frailty than those with 12 pairs or more (Table 3, Model 3).

**Table 3:** Risk Factors of Oral Health Status for Cognitive Frailty Before and After Adjusted Analyses

Characteristics	Model 1	*P*	Model 2	*P*	Model 3	*P*
	OR(95% C.I.)		OR(95% C.I.)		OR(95% C.I.)	
**Tongue**						
Health	1.00	0.037	1.00	0.146	1.00	0.326
Changes	1.28(0.96, 1.71)	0.092	1.06(0.75, 1.48)	0.754	0.96(0.68, 1.36)	0.825
Unhealthy	1.88(1.10, 3.21)	0.021	1.87(0.99, 3.49)	0.051	1.57(0.82, 2.97)	0.171
**Natural teeth**						
Health	1.00	0	1.00	0.006	1.00	0.013
Changes	1.67(1.09, 2.54)	0.017	1.34(0.82, 2.20)	0.240	1.30(0.79, 2.14)	0.309
Unhealthy	3.15(2.12, 4.67)	< 0.001	2.01(1.27, 3.19)	0.003	1.91(1.20, 3.07)	0.007
**Masticatory teeth**						
Health	1.00	< 0.001	1.00	0.002	1.00	0.002
Changes	1.67(1.16, 2.39)	0.006	1.70(1.11, 2.59)	0.014	1.72(1.14, 2.66)	0.014
Unhealthy	3.48(1.94, 6.22)	< 0.001	2.69(1.39, 5.19)	0.003	2.62(1.34, 5.11)	0.005

**Model 1** included the total BOHSE score and 10 items: lymph nodes, lips, tongue, mucous tissue, gums, saliva, natural teeth, dentures, masticatory teeth and oral hygiene;

**Model 2** adjusted for age, gender, education level, living arrangement and BMI;

**Model 3** adjusted for age, gender, education level, living arrangement and BMI, PSQI, diabetes and depression

*OR* Odds ratio, *CI* Confidence interval

## Discussions

The present study demonstrates that old patients with cognitive frailty have worse oral health status compared with non-cognitive frailty. In particular, four decayed or defective teeth or residual roots, and less than 11 pairs of teeth in a normal occlusal relationship in the chewing position were independent risk factors for cognitive frailty. To our best knowledge, this is the first study to report detailed oral health examinations in patients with cognitive frailty over 60 years old.

BOHSE was compiled by Kayser-Jones et al. in 1995 [17]. It has been described as the most comprehensive, effective and reliable oral health screening tool, which is available for utilization by non-dental professionals, regardless of the patient’s cognitive function [18, 19]. The Chinese version of BOHSE is suitable for older population in China and can be used for the evaluation of older patients in hospital. The retest reliability was 0.775, which was similar to the original scale (0.83 − 0.79). The inter-confidence was 0.737, higher than 0.64 − 0.40 of the original scale. So far, no study has reported the oral status of patients with cognitive frailty in detail by using the oral health related assessment scale. We conducted a detailed oral health assessment using the BOHSE scale in patients over 60 years of age with cognitive frailty. We found that the average scores of BOHSE, the proportions of changes, unhealthy states of the tongue, mucosa tissue, gums, natural teeth, dentures, masticatory teeth and oral hygiene in cognitive frailty are higher than those of the non-cognitive frailty patients. At the same time, growing researches suggest that poor oral health in the elderly interacts with frailty, malnutrition, and cognitive decline. By providing appropriate oral health treatment to the elderly population, the age-related functional decline can be offset and the health-related quality of life can be improved. Increasing evidence also reveals it [20]. Therefore, it is necessary to pay attention to the oral health status of patients with cognitive frailty.

So far, only one study has reported that older adults with 20 or more teeth have a lower risk of cognitive frailty than individuals who have less than 20 teeth (OR = 0.66, 95% CI: 0.44–0.99, *P* = 0.046) [16]. But oral problems in patients with cognitive impairment and frailty alone have been widely discussed. One Chinese study found that patients with MCI lost an average of 11.8 teeth, higher than those with normal cognitive function (9.3 teeth), and having over 16 missing teeth was associated with severe cognitive impairment [21]. In a descriptive study of the national database of Japan’s national health care system, patients with poor cognitive function were found to have more missing or toothless teeth, resulting in reduced tooth contact and reduced chewing function [22]. One cross-sectional study indicated that older people with lower anterior tongue movement and lower masticatory performance were positively associated with physical pre-frailty [23]. A study examines the reciprocal relationship between cognitive function and edentulism, they found that among respondents aged 60 or older, baseline cognitive function was associated with subsequent edentulism, and baseline edentulism was also associated with follow-up lower levels of cognitive function [24]. According to a recent meta-analysis, there was a linear association between tooth loss and cognitive impairment or dementia, for example, each additional tooth loss was associated with a 0.014 increased relative risk of cognitive impairment and 0.011 elevated relative risks of dementia. Timely denture repair can reduce the progression of cognitive decline due to tooth loss [13]. Although these studies have identified a reduction in the number of natural teeth as a risk factor for cognitive impairment, it is not clear how much loss of teeth is associated with an increased risk of cognitive impairment. Our study found that one to three teeth decayed or broken was not associated with an increased risk of cognitive frailty, but four or more natural teeth decayed or broken was associated with a 1.91 times greater risk of cognitive frailty. Therefore, it is suggested that patients over 60 years of age with cognitive frailty should receive timely restoration when four teeth are decayed or broken.

Although most studies have found that timely denture repair can improve the cognitive impairment associated with tooth loss, however, not all dentures provide these benefits. Patients who wear complete dentures often complain of issues such as bad adjustment, more limited range of ingestible food, discomfort and dissatisfaction. As a consequence, the cerebral blood flow is reduced and cognitive function is not improved significantly [25]. In our study, we found that a reduction in the number of teeth was a risk factor for cognitive frailty, and a reduction in masticatory counterpoint was also a risk factor for cognitive frailty. For example, at the chewing position, the risk of cognitive frailty was 1.72 to 2.62 times higher for those with normal occlusal relationships of less than 11 pairs than for those with normal occlusal relationships of more than 12 pairs. Another study suggested that both the retention of natural teeth and the rehabilitation of the missing teeth with prostheses and guaranteeing functional denture quality could attenuate cognitive impairment. Animal and human experiments have also shown that activation of masticatory muscles and normal chewing can induce the release of several mediators and activation of specific brain regions, jointly leading to higher neuronal activity, neurotrophic support, blood flow, and prevention of amyloid plaque formation [26]. Therefore, as for improving oral health, we should not only pay attention to the number of teeth restoration, but also take into account the restoration of chewing function of teeth.

Although the mechanism for the worse oral health in cognitive frailty group found in the present study is still unknown, it can be inferred that the multiple factors may be plausible explanations as the mechanism mediating the association between oral unhealthy status and cognitive frailty. First, chronic inflammatory process may be involved in the development of cognitive frailty and tooth loss. Previous studies have suggested that chronic inflammatory stimulation is involved in the pathophysiological mechanism of cognitive frailty [27]. In addition, the most important reason for tooth loss in older individuals is the long-term cumulative effects of periodontitis [28]. Oral pathogens and their toxic molecules, after disseminating into the bloodstream, may induce a low-grade systemic inflammation through upregulating the release of cytokines and inflammatory mediators, which can trigger neuroinflammation and cause neuronal degeneration. A recent study identified a pathogen (porphyromonas gingivalis) as a key cause of chronic periodontitis in the brains of patients with Alzheimer’s disease and confirmed that chronic inflammation in the mouth could affect cognitive function [29, 30]. Cognitive frailty and periodontitis are both related to inflammation. Oxygen-Ozone treatment has a unique anti-inflammatory effect. Although Oxygen-Ozone therapy can be theoretically utilized for cognitive frailty and oral inflammation, further studies are needed in clinical practice [31, 32]. Second, decreased chewing function may result in cognitive impairment. Generally speaking, the effective occlusion is closely related to the number of teeth. The loss or damage of tooth will inevitably lead to the reduction of the number of occlusal tooth and subsequently the reduction of chewing function. Recently, it has been found that masticatory muscles respond to cholinergic stimuli during chewing by retrograde delivery of exosome Neprilysin (NEP) to the brain, bypassing the blood-brain barrier, which helps to reduce the burden of Aβ plaques in the brain, thereby improving cognitive function [33]. In another investigation, during chewing, cortical blood flowing to the somatosensory cortex regions increases, and oxygen levels in the prefrontal cortex and hippocampus increase. On the contrary, reduced effective chewing leads to decreased cerebral blood flow, impaired spatial memory and hippocampal neuron degeneration, which can increase the risk of cognitive decline.

Our study found that patients with cognitive frailty had poorer oral health. More than four natural teeth decayed or broken and fewer than 11 pairs of teeth in chewing position were associated with an increased risk of cognitive frailty. Therefore, in the clinical settings, it is necessary to pay more attention to the oral health status of patients with cognitive frailty in the screening and management processes. At the same time, with regards to restoration of oral health function, we should not only do the restoration of the number of teeth, but also need to take into account the restoration of tooth counter position and chewing function.

There are some limitations in our study. First of all, because our study was a cross-sectional study, it would hamper causal inference between the worse oral health status and thereafter cognitive frailty. Subsequently, prospective cohort studies should have been conducted to explore the impact of oral health problems on the occurrence and development of cognitive frailty. Therefore, attention should also be paid to whether oral health interventions have positively preventive or therapeutic effects on cognitive frailty. Finally, we found that more than four natural teeth decayed or broken and reduced masticatory teeth were major oral health problems in cognitive frailty patients, but did not further explore the mechanisms. It is necessary to explore further studies in the follow-up research.

## Conclusion

In the present study, we comprehensively assessed the oral health status of older hospitalized patients over 60 years of age complicating with cognitive frailty. Four or more natural teeth decayed or broken and a reduction in chewing pairs were predominantly independent risk factors for cognitive frailty. Such a positive result suggests that it is necessary to carry out oral health examination for older patients with cognitive frailty. Repairing missing teeth in time and masticatory counter position improvement may be the effective methods for early intervention for cognitive frailty.

## Data Availability

The datasets used and analyzed during the current study are available from the corresponding author on reasonable request.

## Abbreviations

BOHSE: Brief Oral Health Status Examination
BMI: Body Mass Index
PSQI: Pittsburgh Sleep Quality Index
